# On the Use of Biaxial Properties in Modeling Annulus as a Holzapfel–Gasser–Ogden Material

**DOI:** 10.3389/fbioe.2015.00069

**Published:** 2015-06-03

**Authors:** Narjes Momeni Shahraki, Ali Fatemi, Vijay K. Goel, Anand Agarwal

**Affiliations:** ^1^Mechanical, Industrial and Manufacturing Engineering Department, University of Toledo, Toledo, OH, USA; ^2^Engineering Center for Orthopaedic Research Excellence, University of Toledo, Toledo, OH, USA; ^3^Bioengineering Department, University of Toledo, Toledo, OH, USA

**Keywords:** functional spinal unit, finite element modeling of intervertebral disk, annulus material modeling, hyperelastic and anisotropic behavior, uniaxial vs. biaxial stress state

## Abstract

Besides the biology, stresses and strains within the tissue greatly influence the location of damage initiation and mode of failure in an intervertebral disk. Finite element models of a functional spinal unit (FSU) that incorporate reasonably accurate geometry and appropriate material properties are suitable to investigate such issues. Different material models and techniques have been used to model the anisotropic annulus fibrosus, but the abilities of these models to predict damage initiation in the annulus and to explain clinically observed phenomena are unclear. In this study, a hyperelastic anisotropic material model for the annulus with two different sets of material constants, experimentally determined using uniaxial and biaxial loading conditions, were incorporated in a 3D finite element model of a ligamentous FSU. The purpose of the study was to highlight the biomechanical differences (e.g., intradiscal pressure, motion, forces, stresses, strains, etc.) due to the dissimilarity between the two sets of material properties (uniaxial and biaxial). Based on the analyses, the biaxial constants simulations resulted in better agreements with the *in vitro* and *in vivo* data, and thus are more suitable for future damage analysis and failure prediction of the annulus under complex multiaxial loading conditions.

## Introduction

Intervertebral disks are interposed between the two adjoining vertebral bodies along the spine. They impart stability and flexibility to the human spine. A disk comprises three different components: annulus fibrosis (AF), nucleus pulposus (NP), and cartilaginous endplate (EP). NP is the central part of disk enclosed in the annulus and bonded to superior and inferior cartilaginous EPs. The water content of the hydrated NP is 90% at birth. It decreases with age to 80% at 20 years and 70% by 60 years and beyond as a part of the aging process (Iatridis et al., 1996). The change in the water concentration with age may lead to the disk degeneration.

Intervertebral disks are one of the primary sources of acute low back pain because of disk degeneration and disk herniation (Kuslich et al., 1991). However, the underlying mechanisms for the disk degeneration are still unclear, especially the relationship between the external loads and damage to disk annulus. Magnetic resonance images (MRI) show annular tears, mostly in the lumbar intervertebral disks and especially at the L4–L5 level (Rajasekaran et al., 2013).

Three types of tears are prevalent: radial, circumferential, and the rim lesions (Osti et al., 1992; Vernon-Roberts et al., 2007). These tears or cracks can be produced by combinations of several loads experimentally. For example, an *in vitro* model with physiological stress produced under a cyclic combination of compression, rotation, and flexion supports that annular protrusion occurs in combined loading condition, and AF is the site of primary pathologic changes (Gordon et al., 1991). In another experiment, the effect of combination of flexion and torsion on the ovine lumbar disk herniation has been studied. The results of this study indicate that this combination effectively increased the appearance of radial tear in the AF (Veres et al., 2010).

To investigate the initiation location and then the propagation of cracks in the intervertebral disk, we need to have a good understanding of the stresses and strains in the intervertebral disk. Finite element analysis (FEA) is one of the most effective tools to evaluate the internal response of the disk due to external loads, since it is difficult, if not impossible, to experimentally measure stresses under several loading conditions. Stress and strain distributions obtained from such analysis depend on the geometry, material model, and material properties used in the simulations. For example, modeling geometry varies from disk alone to full ligamentous functional spinal unit (FSU) simulations (Natali, 1991; Kumaresan et al., 1999; Rohlmann et al., 2006; Schroeder et al., 2010). Different material models and techniques are also used to simulate each component of the intervertebral disk. Nucleus varies from linear and non-linear elastic to fluid in the simulations (Wang et al., 1997; Yao et al., 2006). Different techniques are also used for AF modeling.

Layer by layer investigation of the human AF structure using microscopic techniques has shown that AF consists of 15–25 distinct layers, depending on spine level and age (Marchand and Ahmed, 1990). The collagen fibers have two defined orientations that change from one layer to the next. The typical angle of fibers has been reported to have a wide range (Marchand and Ahmed, 1990), with an average of ±30° with respect to the transverse plane implemented in some previous studies. Tensile properties of the human AF from single and multiple lamellae samples suggest non-linear and anisotropic behavior of this tissue (Green et al., 1993; Skaggs et al., 1994; Ebara et al., 1996; Fujita et al., 1997; Holzapfel et al., 2005).

Founded on the AF complex structure, different material models have been proposed to represent this structure. Mixture of fibers and matrix in a composite structure with linear and non-linear properties of fibers and matrix has been a commonly used modeling technique (Shirazi-Adl et al., 1984; Lu et al., 1996; Wang et al., 1997; Ferguson and Steffen, 2003; Schmidt et al., 2006; Little et al., 2007). In addition, the roles of porosity, permeability (Natarajan et al., 2007; Galbusera et al., 2011; Qasim et al., 2012), or viscoelasticity (Schroeder et al., 2006) have also been investigated. In these models, fibers are modeled using rebar or spring elements and anisotropy is introduced by specifying their orientation. Modeling of such composite structures is, however, highly dependent on model parameters, which are often difficult to determine (for instance, fiber and matrix properties, or fiber volume fraction). These models have extensively been used for spine kinematics under various loading conditions (Rohlmann et al., 2009; Dreischarf et al., 2014). Failure analysis based on these classical models has been conducted using ultimate tensile strain in the fibers (Shirazi-Adl, 1989) and also stresses in the ground substance (Qasim et al., 2012).

A model, which adequately describes disk mechanics and stress distribution under multiaxial loading, is essential for annulus damage and failure analysis. In the present study, because of paucity of a clear picture of clinically observed annulus damage vis-à-vis the mechanical loads, we have used a hyperelastic anisotropic material model for the AF to investigate the biomechanical behavior of the ligamentous L4–L5 segment. This material model was initially proposed for arterial layers (Holzapfel et al., 2000) and is based on the strain energy function described in Spencer (1984). Later, this material model has been used by various investigators for different purposes such as modeling intervertebral disk (Eberlein et al., 2001). In this material model, hereafter referred to as Holzapfel–Gasser–Ogden (HGO) model, AF is presented as a continuum material and the effects of fibers and matrix are considered in the strain energy. Strain distributions from hyperelastic anisotropic material model of AF were compared to a model based on classical method. Unexpected strain concentrations in the posterior part of the matrix were observed in the classical material model based on a composite structure with fibers and matrix, but not in the HGO material model (Eberlein et al., 2001). These concentrations were explained to be owing to two deficiencies of the classical material model: matrix considered as linear elastic, and fibers considered in short-fiber arrangement.

The hyperelastic anisotropic continuum model has been used to study the kinematics of the lumbar spine (Del Palomar et al., 2008; Moramarco et al., 2010). However, uniaxial properties of the AF were used in those studies. By contrast, *in situ* conditions that AF experiences is a biaxial stress state. Therefore, based on experimental data for this material model obtained from uniaxial and biaxial tests, the aim of this paper is to highlight the biomechanical differences [e.g., intradiscal pressure (IDP), motion, and stresses] resulting from dissimilarities between the two sets of material properties (uniaxial and biaxial). It is shown that the material properties from biaxial tests simulate the annulus fibrosus with hyperelastic anisotropic material model more accurately than properties from uniaxial tests and are more relevant to the *in situ* condition. A discussion and analysis of model properties relevant to understanding clinically relevant disk mechanics under different loading conditions is also presented. A model, which adequately describes disk mechanics and stress distribution under such complex loading, is essential for AF damage and failure analysis.

## Materials and Methods

### Finite element model

**Figure 1:**
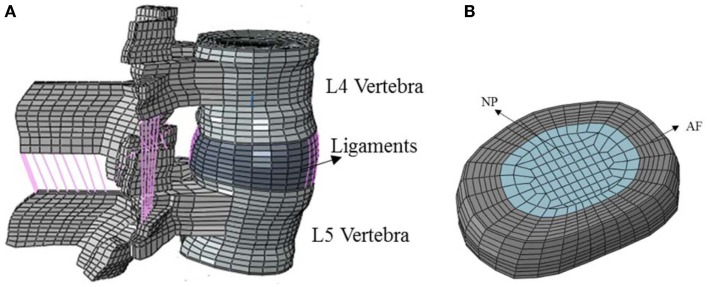
**(A)** Ligaments in the FE model of the functional spinal unit, **(B)** FE model of intervertebral 539 disc consisting of AF and NP.

A previously valid three-dimensional (3D) FE model of ligamentous L4–L5 FSU (Goel et al., 1995) was used. This model is one of the well-established models, which has been used for modeling lumbar spine (Dreischarf et al., 2014). The geometry was developed from computer tomography (CT) scans. The slice thickness was approximately 1.5 mm and the thickness of the cortical layer was 0.5 mm across the model. The model was validated using *in vitro* kinematic data, facet loads, ligaments strains, and disk bulge under several loading conditions (Goel et al.gray, 1995gray, 2007; Dooris et al., 2001). The model consists of cortical and cancellous vertebral bones, posterior bony elements, annulus, nucleus, facet joints, and seven major ligaments, Figure 1A. This model contains 15,136 elements and 19,148 nodes and is symmetric with respect to the mid-sagittal plane (Monore, 1993; Kiapour et al., 2012; Dreischarf et al., 2014). For bony structure and intervertebral disk components, C3D8 brick elements were used. All of the ligaments were modeled using two nodes tension-only truss elements (T3D2) with non-linear material properties.

Intervertebral disk was divided in two separate anatomical parts: AF and NP, the same as the previous model, Figure 1B. Linear isotropic material constitutive relationship was used in NP region and a hydrostatic pressure was simulated. Several approaches have been used to simulate the incompressibility of the nucleus, including the one used in the present study. All of the approaches have yielded clinically relevant results. This approach for modeling has been well established. We have also used a hyperelastic neo-Hookean model for the annulus and incompressible fluid simulation for the nucleus. The results were similar. The range of elasticity modulus that has been considered for NP in the literature is within 1.5–10 MPa (Wang et al., 2000; Chen et al., 2001, 2008; Ayturk and Puttlitz, 2011) and here it was assumed to be 2 MPa. AF complex structure was modeled by the hyperelastic and anisotropic material model of HGO, which is available in ABAQUS/Standard™ version 6.10 [Simulia Inc, Rhode Island-USA]. The facet joints had a gap of 0.5 mm and could only transmit compressive forces. Facet contact stiffness was modeled with a non-linear exponential function with GAPUNI elements. The elastic properties of the different parts are listed in Table 1.

**Table 1:** **Material properties and element type of bony structures, ligaments, intervertebral disk, and facet joint (Agarwal et al., 2013a,b)**.

Material	Element type	Constitutive relation	Material properties
**Bony structures**			
Vertebral cortical bone, endplates, and posterior cortical bone	8 Nodes brick element (C3D8)	Isotropic, elastic	*E* = 12,000 MPa ν = 0.3
Vertebral cancellous bone and posterior cancellous bone	8 Nodes brick element (C3D8)	Isotropic, elastic	*E* = 100 MPa ν = 0.2
**Ligaments**			Elastic modulus MPa (strain %)
Anterior longitudinal	Tension-only, truss elements (T3D2)	Hypoelastic	7.8 (<12%), 20.0 (>12%) ν = 0.3
Posterior longitudinal	Tension-only, truss elements (T3D2)	Hypoelastic	10.0 (<11%), 20.0 (>11%) ν = 0.3
Ligamentum flavum	Tension-only, truss elements (T3D2)	Hypoelastic	15.0 (<6.2%), 19.5 (>6.2%) ν = 0.3
Intertransverse	Tension-only, truss elements (T3D2)	Hypoelastic	10.0 (<18%), 58.7 (>18%) ν = 0.3
Interspinous	Tension-only, truss elements (T3D2)	Hypoelastic	10.0 (<14%), 11.6 (>14%) ν = 0.3
Supraspinous	Tension-only, truss elements (T3D2)	Hypoelastic	8.0 (<20%), 15.0 (>20%) ν = 0.3
Capsular	Tension-only, truss elements (T3D2)	Hypoelastic	7.5 (<25%), 32.9 (>25%) ν = 0.3
**Intervertebral disk**			
Nucleus pulposus	8 Nodes brick element (C3D8)	Isotropic, elastic	*E* = 2 MPa ν = 0.499
Annulus fibrosus	8 Nodes brick element (C3D8)	Hyperelastic anisotropic (HGO)	Table 2
**Apophyseal joints**	GAPUNI elements	Non-linear soft contact	Pressure overclosure = 12,000 MPa

### Annulus material model

The annulus was modeled in a different manner, as opposed to fibers embedded in a ground substance in the classical model, as explained in the Section “Introduction.” The HGO material model is based on the strain energy potential for distributed collagen fibers in the ground substance (Holzapfel et al., 2000; Gasser et al., 2006). The primary advantage of using this material model rather than phenomenological models is that the fibers and matrix material properties are associated with the material constituents (histological structure). In addition, the material parameters for the model can be experimentally determined, as described earlier.

In this material model, exponential material behavior for fibers and non-linear hyperelastic neo-Hookean isotropic material model for the ground substance were used. Unlike previous modeling that considered discontinuous arrangement for annulus fibers, in HGO material model collagen are arranged as continuous fibers. These models for the matrix and fiber are represented by Eqs 1 and 2, respectively:

(1) $$W_{\text{matrix}}=C_{10}\left(\bar{I_{1}}-3\right)+\frac{1}{D}\left(\frac{J^{2}-1}{2}-\ln J\right)$$

(2) $$W_{\text{fiber}}=\frac{K_{1}}{2K_{2}}\sum_{\alpha =1}^{N}\left\{\exp\left(K_{2}\left(\bar{E_{\alpha }^{2}}\right)-1\right)\right\}$$

with:

(3) $${\bar{E}}_{\text{α}}=\text{κ}\left({\bar{I}}_{1}-3\right)+\left(1-3\text{κ}\right)\left({\bar{I}}_{4}\left(\alpha \alpha \right)-1\right)$$

where, ${\bar{I}}_{1}$ is the first deviatoric strain invariant, *J* is the elastic volume ratio, and ${\bar{I}}_{4\left(\text{αα}\right)}$ is pseudo-invariants of the distortion part of the right Cauchy–Green strain and unit vectors in the direction of fiber families. Matrix compressibility and matrix stiffness are defined by *D* and *C*_10_ in Equation 1, respectively, *K*_1_ > 0 in Eq. 2 is a material parameter with a dimension of stress and relates to the stiffness of fibers. *K*_2_ > 0 in this equation is a dimensionless material property that is related to fiber non-linear behavior. Fibers in this structural material model can support only tensile stresses, therefore, Eq. 2 applies in fiber extension mode (Gasser et al., 2002). *N* is the number of fiber families in Eq. 2. In the AF, there are two fiber families with ±30° orientation with respect to the horizontal plane. Value of κ in Eq. 3 is between $\frac{1}{3}$ for the randomly oriented fibers and 0 for aligned fibers. Here, we considered the two fiber families of AF to be aligned fibers for simplicity.

For incompressible materials, *D* is assumed to approach infinity. Because we can assume the matrix as an incompressible material (Natali, 1991), the second terms of the matrix formulation are eliminated. Only three material constants (*C*_10_, *K*_1_, and *K*_2_) are needed. The equation for strain energy potential will then simplify as Eq. 4. The values of these three constants for both uniaxial tension (O’Connell et al., 2009) and biaxial tension (O’Connell et al., 2012) stress states are given in Table 2.

(4) $$W=C_{10}\left({\bar{I}}_{1}-3\right)+\frac{K_{1}}{2K_{2}}\sum_{\text{α}=1}^{2}\left\{\exp\left(K_{2}{\left({\bar{I}}_{4}(\text{αα})-1\right)}^{2}-1\right)\right\}$$

**Table 2:** **Uniaxial tension and biaxial tension parameters for HGO material model**.

Holzapfel–Gasser– Ogden parameters	Uniaxial values (O’Connell et al., 2009)	Biaxial values (O’Connell et al., 2012)
*C*_10_	0.035 MPa	0.85 MPa
*K*_1_	0.296 MPa	2.8 MPa
*K*_2_	65	90

It should be noted that while hyperelastic neo-Hookean isotropic material with the associated material constant *C*_10_ is used as the material model for the ground substance, the material model used by O’Connell et al. (2012) from which the value of *C*_10_ was taken is the Mooney–Rivlin model. However, with assuming an incompressible material as mentioned previously, the value of constant *C*_10_ is identical in both the neo-Hookean and Mooney–Rivlin models.

As can be observed from Table 2, there is a significant difference between the biaxial parameters and uniaxial parameters. This is mentioned in the literature as well (Gregory and Callaghan, 2011). In this work, we used both sets of parameters in two separate 3D finite element models of FSU that were previously described. It was desired to evaluate and compare FSU kinematics and stress behavior in response to this change to determine which set of material properties produce more accurate results and are more suitable for damage analysis in future.

### Boundary and loading conditions

The inferior surface of the L5 vertebra was rigidly fixed. Compressive follower loads were applied up to 3400 N. Using follower loads in a motion segment, the compressive load is tangent to the spinal curve and, therefore, intervertebral disk would be loaded in almost pure compression. The predicted average load vs. axial displacements in the anterior and posterior regions of the disk were compared to the *in vitro* experimental data in which pure axial compression loads were applied only to the intervertebral disk (Markolf and Morris, 1974). Therefore, both FE analysis and experiments would produce the same loading scenario in the intervertebral disk (pure compression) for comparison.

The annulus axial stresses along antero-posterior direction at the middle disk plane for 2000 N were compared to the experimental data (Adams et al., 1996). They also indicated that extension caused the apophyseal joints to become load-bearing, and damage could occur at compressive loads as low as 500 N (Adams et al., 1994). The models were also subjected to compressive forces of 300, 700, 850, and 3400 N simulating equivalent compressive loads on the supine, standing, walking, and lifting 44 lb with back bent and knees straight positions, respectively (Nachemson, 1966, 1975, 1991).

The IDP was defined as the hydrostatic pressure [−1/3*tr(σ), where tr(σ) is the first invariant of the stress tensor σ] in the center of the nucleus, and was compared with available *in vivo* results (Wilke et al., 1999). However, the results of these studies are presented for different spinal levels and may not be directly comparable. Nachemson (1966) introduced a correction factor for estimation of the compressive force. The problem of the estimation of compressive loads has been discussed in Dreischarf et al. (2013). Pure moments in different directions on the L4–L5 level were also applied. The axial displacement, flexion, extension, lateral bending, and axial rotation values for the aforementioned loads/moments were computed for comparison with data reported in the literature and evaluation of the hypothesis.

The range of motion for different loading conditions for the two FE models used in this study are compared to experimental data from Agarwal et al. (2013c). For these experiments, spine segments with ligaments attached were used. Each specimen was subjected to pure moments using rods and pulleys to simulate flexion, extension, lateral bending, and axial rotation. The spatial coordinate data obtained were used to compute the 3D segmental rotations.

## Results

**Figure 2:**
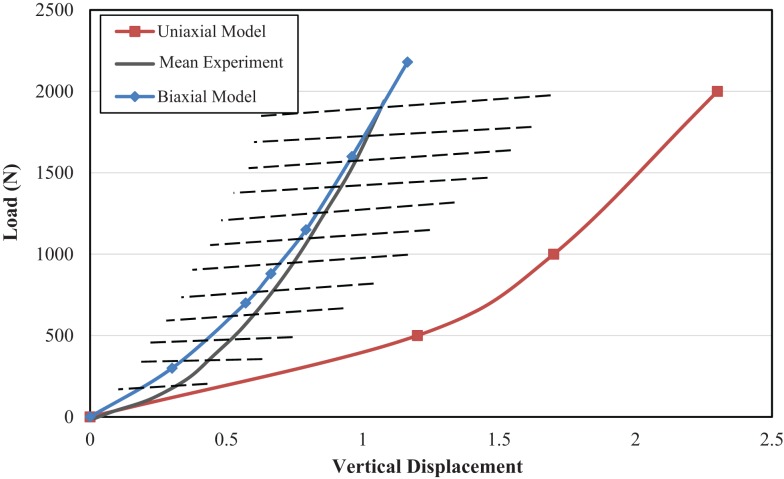
**Comparison of load-displacement curves between uniaxial and biaxial models and as compared to experimental results from Markolf and Morris (1974) for compressive force applied as follower load to the L4–L5 level**. Dash lines indicate the experimental range.

**Figure 3:**
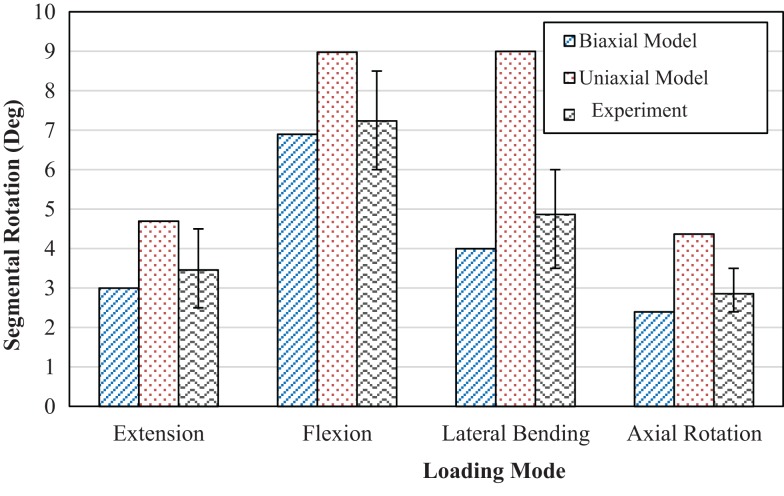
**Comparison of range of motion between models with uniaxial and biaxial material properties with experimental results from Agarwal et al. (2013c) at 10 Nm pure moment in several directions**.

The mean and range of load vs. axial displacement for an *in vitro* experiment (Markolf and Morris, 1974) on healthy intervertebral disk only, and the results of current FE study of both models with uniaxial and biaxial material properties are presented in Figure 2. The predicted load-axial displacement of the model with biaxial material properties is between the experimental ranges. However, load-axial displacement for the uniaxial model is below the lower limit of the experimental data. Range of motion for different loading conditions for the two FE models are compared to data from Agarwal et al. (2013c) in Figure 3 and indicate the results from the FE model based on biaxial properties are closer to the experimental data.

The outcomes shown in Figure 3 indicate that the model with uniaxial material properties resulted in higher values in extension by 57%, flexion by 30%, axial rotation by 82%, and lateral bending by 125%, compared to the model with biaxial material properties. In flexion, extension, and lateral bending, the results of the predicted biaxial model based data were within the SD of the experimental data. Axial rotation data for the biaxial model were close to the lower limits of the experimental data. However, the corresponding data for the uniaxial material properties for all loading conditions exceeded the upper limit of the experimental data, Figure 3.

**Figure 4:**
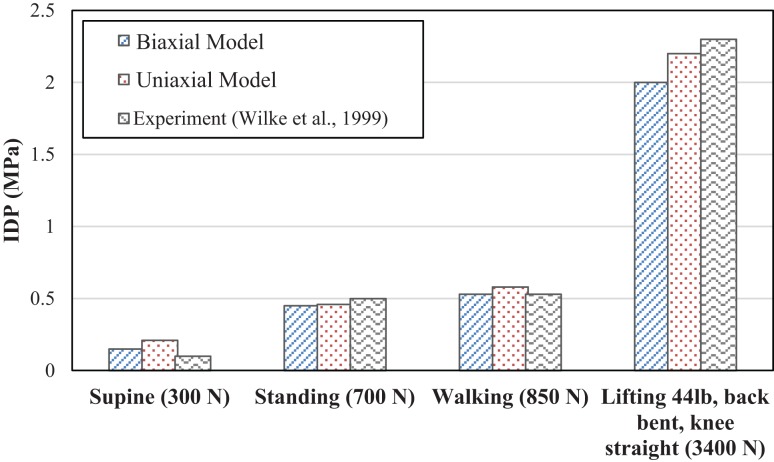
**Comparison of intradiscal pressure (IDP) between simulations with uniaxial and biaxial material property models as compared with mean values of experimental results (Wilke et al., 1999) under the same loading conditions**.

**Figure 5:**
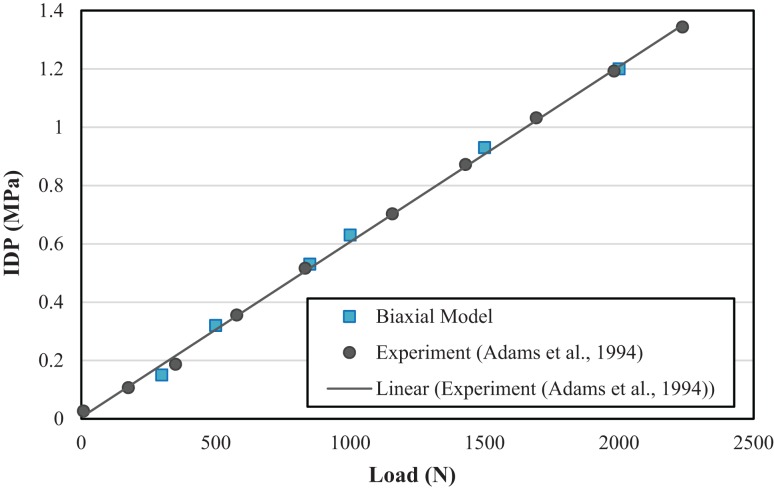
**Comparison of intradiscal pressure (IDP) with FE model with biaxial material properties and experimental results from Adams et al. (1994)**.

Corresponding IDPs for the biaxial and uniaxial models vs. the *in vivo* experimental data for various compressive forces are plotted in Figure 4. The difference between these two models (uniaxial vs. biaxial) was <10%. Both of the models show close results to the experimental data (Wilke et al., 1999). Figure 5 shows a more detailed picture of IDP for biaxial-based FE model vs. applied loads and as compared to experimental data with the same loading conditions (Adams et al., 1994).

**Figure 6:**
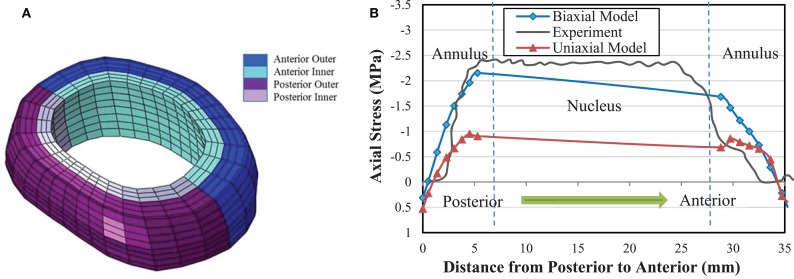
**(A)** Different anatomical regions in the FE model of AF. **(B)** Axial stress distribution obtained from biaxial and uniaxial FE models in the mid-height plane of intervertebral disk from posterior to anterior midline and comparison with the experimental data from McNally and Adams (1992) at 2000 N compressive force.

Axial stress distribution along the anterior–posterior direction (Figure 6A) in the middle disk height plane for 2000 N compression in the AF is shown in Figure 6B. The difference between the model results with biaxial properties and experimental data (McNally and Adams, 1992) is 25%. However, this difference in the uniaxial model is 60%. Thus, the model based on the biaxial material properties in both cases exhibited closer trend to the experimental results, as compared with the model based on uniaxial properties.

**Figure 7:**
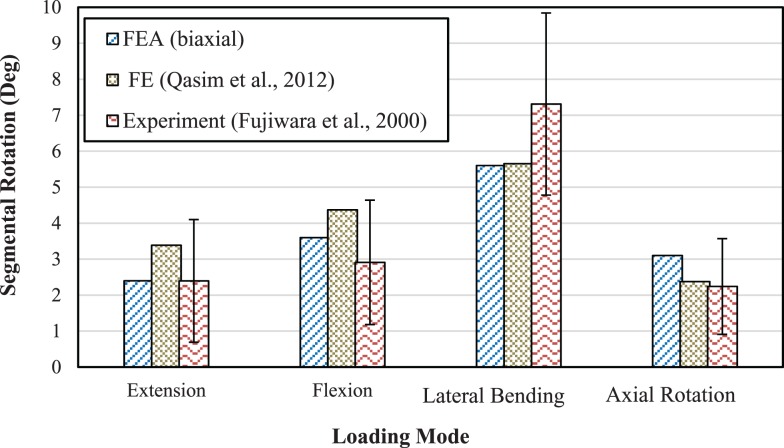
**Comparison of range of motion between the model with biaxial material properties and the detailed FE model from Qasim et al. (2012) as well as with experimental results from Fujiwara et al. (2000) for 6.6 Nm moment**.

To compare the results in this work with an example of a study using a continuum damage mechanics methodology based on a composite model with fibers and matrix, the results from Qasim et al. (2012) were considered. Our prediction based on biaxial model with no refinement for porosity and other parameters are in agreement with the results from this study, Figure 7.

## Discussion

The aim of the current study was to use an anisotropic material model for the AF region and compare the predicted FSU biomechanics based on using uniaxial or biaxial material properties assigned to the AF. The predicted data were also compared to relevant experimental data from the literature. These experiments were among the most accepted and cited experiments in the field.

The load vs. axial displacement results obtained from biaxial material properties showed good agreement with experimental data (Figure 2). Uniaxial properties led to more axial deformation, in comparison to the biaxial material properties. This lower stiffness is also observed in the angular rotation under applied moments in several directions (Figure 3). The higher stiffness of the motion segment with biaxial material properties of AF led to higher axial stresses in the AF, much closer to the experimental data (Figure 6B). This indicates that biaxial material properties represent a stress state closer to the *in situ* condition. In addition, *in situ* AF fibers are attached to the upper and lower vertebral bodies, which provide a constrained condition. Therefore, the stress state of AF *in situ* condition is in agreement with biaxial tests. This condition has also been suggested in similar studies on other soft tissues such as skin, when subjected to *in vivo* multi axial stress state (Tong and Fung, 1976), and arteries (Debes and Fung, 1995).

Non-linear behavior of the lumbar segment under axial compressive force, which has been observed in experiments (Kulak et al., 1976) was predicted by non-linear material model used for AF region for both the uniaxial and biaxial material property models. Experiments have shown that IDP has a linear relationship in pure compression force (Adams et al., 1994), and our predictions are in agreement for both uniaxial- and biaxial-based models, Figure 4.

Instead of considering separate stresses in the matrix and fibers, a combined nominal stress as obtained in this study for AF gives the opportunity to compare the model results directly with experimental data. Axial stress distribution resulting from compressive force leads to a peak stress on the posterior region of the disk according to the model results, Figure 6B. Posterior region is the area that has the most likelihood of damage, according to clinical data (Vernon-Roberts et al., 2007). Tensile stresses in outer annulus regions observed in Figure 6B may be regarded as contribution of the annulus fibers to the total stress tensor, which is not measurable experimentally.

It was shown that incorporating biaxial material properties in a hyperelastic anisotropic material model result in internal stresses in the intervertebral disk that are in agreement with the *in vitro* data, as compared with the properties obtained from uniaxial tests. The magnitude of stresses has a dominant effect on damage evaluation and failure prediction of AF. Further work is being pursued to document damage prediction using the biaxial material property model.

To develop uniaxial- and biaxial-based models, experimental data from other studies were used and their limitations, highlighted in those studies, will be reflected in our model as well. In these experimental studies, the properties were calculated for outer anterior site of AF (O’Connell et al., 2009, 2012). Therefore, we used the same data for the model used in this current study as a homogenous model. In the experimental studies, degeneration effects have been considered as well. It has been shown that for biaxial stress state matrix parameters (*C*_10_ and *D*) and fiber stiffness (*K*_1_) in Eq. 4 do not correlate with degeneration, whereas fiber non-linearity (*K*_2_) decreases with increasing grade of degeneration (O’Connell et al., 2012). However, for uniaxial stress state matrix parameters contribute to represent the effect of degeneration (O’Connell et al., 2009). In the current study, we focused on the healthy AF properties, but the models can be modified to account for changes in parameters as a function of degeneration.

Qasim et al. (2012) refined a basic model by adding properties like porosity, strain dependent permeability, and osmotic pressure into their model. Agreement of the results in this study without refinement for porosity and other parameters with those from Qasim et al. (2012), Figure 7, suggests that effects of porosity and other parameters such as water content may not significantly change the findings due to the static and short-time loading conditions that were considered in this study. For more robust model predictions and for long-time loading conditions, however, these refinements should be included to evaluate their effects, as they contribute to viscous behavior. The loading rate has also been shown to greatly influence mechanical response of spinal structures to external loads (Ochia et al., 2003). Therefore, viscoelasticity, which is a characteristic of soft tissues, can affect stress and strain distributions and is important for damage analyses and failure prediction. Although incorporation of this effect into material modeling is very complex, it may need to be considered for higher levels of accuracy.

The annulus region was considered as a homogeneous material in term of fiber orientation and density. Even though the local variations of the fiber orientation were not considered, the model based on biaxial material properties predicts stresses close to those measured experimentally. For more accurate analyses, however, it is necessary to consider this heterogeneous property of annulus.

## Conclusion

This study highlighted the biomechanical differences resulting from dissimilarities between two sets of material properties, uniaxial and biaxial. It was shown that the material properties from biaxial tests simulate the annulus fibrosus behavior more closely, as compared with the experimental data, than properties from uniaxial tests. The predicted load-axial displacement curve of the model with biaxial material properties was shown to be between the experimental ranges, while it is below the lower limit of the experimental data for the uniaxial model. Range of motion for different loading conditions (compression, extension, flexion) from the FE model based on biaxial properties was also shown to be closer to the experimental data. Under a compressive force, axial stress distribution based on the biaxial material properties exhibited closer trend to the experimental results. It is, therefore, concluded that simulation of AF mechanical behavior with biaxial material properties may yield better predictions than with uniaxial material properties under different loading conditions.

## Conflict of Interest Statement

The authors declare that the research was conducted in the absence of any commercial or financial relationships that could be construed as a potential conflict of interest.
